# Experience of Mass Intrahospital Neonatal Transport: Impact on Vitals

**DOI:** 10.7759/cureus.22785

**Published:** 2022-03-02

**Authors:** Rakesh Kumar, Sanober Wasim, Girish Gupta, Suraj Mathur, Damanjeet s Kahlon

**Affiliations:** 1 Pediatrics, Himalayan Institute of Medical Sciences, Swami Rama Himalayan University (SRHU), Dehradun, IND; 2 Neonatology, Himalayan Institute of Medical Sciences, Swami Rama Himalayan University (SRHU), Dehradun, IND

**Keywords:** pretransport stabilisation, intrahospital transport, transport, neonate, neonatal transport

## Abstract

Background

Mass transport of neonates is required in cases of disasters and calamities such as fire. It may also be required when there is a need for the upgradation of infrastructure. Neonatal transport even for a short period is a period of stress for the neonate. Mass transport of neonates needs much planning, and even after diligent planning, may result in the destabilization of neonates.

Objectives

The objectives of this study were to assess the impact of mass intrahospital neonatal transport on the vital parameters of neonates, the occurrence of any adverse event during transport. To study the frequency of adverse events in mass intrahospital neonatal transport and factors related to it.

Materials and methods

This was a retrospective observational study on a cohort of 16 neonates who were transported to an alternate site in the hospital so that renovation and upscaling of the infrastructure of the newborn intensive care unit (NICU) could be undertaken. Site selection, preparation, and transport details were observed. Vital parameters pre and post-transport were noted, and the occurrence of adverse events during transport was documented and analyzed.

Results

Sixteen neonates were transported over a span of 90 minutes with a mean travel time of 5.62±3.03 minutes. There was a statistically significant rise in the heart rate of the neonates post transport (137.7±8.51vs 141.3±9.01, p-value .00769) though not clinically significant. Six point two-five percent (6.25%) of neonates deteriorated post transport and needed extra efforts for stabilization. Equipment malfunction was responsible for deterioration.

Conclusion

Unforeseen events can occur during neonatal transport. Despite adequate planning, preparation, and care during transport, it remains a period of stress for a neonate.

## Introduction

Neonatal transport is the process of shifting a neonate from one setting or facility to another so that a required level of care or type of service that is not available at the former site can be provided [1]. Intrahospital transport of neonates is undertaken for various diagnostic or treatment modalities, which may not be available in the premises of the neonatal intensive care unit (NICU). Common reasons for the intrahospital transport of a neonate are echocardiography and radiology services [2]. Though this happens for a short duration and under safe settings, the neonate remains at risk of developing hypothermia and increased respiratory support [3]. Interhospital transfers are usually of longer duration and are for availing better neonatal services and care. Prior planning and preparation are of paramount importance and greatly impacts the stability of newborn during transport.

Neonatal transport is a time of stress for the neonate, parents, and treating or transporting team, as there are chances of destabilization during transport. Despite the high frequency of intrahospital transport, there is a dearth of data on similar subjects. Studies on mass intrahospital transport of neonates are almost non-existent. We recently had the experience of mass intrahospital transportation of neonates for the renovation and upscaling of the infrastructure of the NICU. The whole process was documented to study the impact of mass transport on neonates.

## Materials and methods

This was a retrospective analysis of a cohort of 16 neonates, admitted to the NICU at the time of shifting, done in a tertiary care teaching hospital in Uttarakhand on December 16, 2020. The NICU was planned for an infrastructure upgrade. To provide continuity of service, it was decided to relocate the neonates to an alternate site while construction work in the present site continues. The department in charge was authorized for the transfer and monitored the process. All neonates admitted in the NICU at the time of shifting were included in the study. All neonates were followed up till the outcome of either discharge, leave against medical advice (LAMA), or death. Data were entered in Microsoft Excel (Microsoft Corporation, Redmond, WA), and IBM SPSS version 25 (IBM Corp., Armonk, NY) was used for analysis. Descriptive statistics were expressed as mean, range, and percentage. Vital parameters were checked for normality by the Shapiro-Wilk normality test. Parametric data were analyzed by the paired t-test and non-parametric data were analyzed using the Wilcoxon matched-pairs signed-rank test.

Prior planning

1. Identification of a Relocation Area

A unit on the same floor as the existing NICU was identified, which was near the labor room and operating theaters so that the distance traveled and the future transfer of neonates requiring NICU care is not affected. The floor space was equivalent to the existing NICU so that all of the beds and pieces of equipment could be stationed comfortably. Because the proposed relocation site was functional with already admitted patients, they were shifted to an adjacent ward, which was closed for care because of the fewer patients due to the coronavirus (COVID) pandemic. An area on the same floor was identified for shifting the beds from the proposed site and all non-required beds were shifted out (n= 40). The area was frequently visited to plan the layout and division of space. The following spaces were earmarked and created: a) Main NICU with warmers and other equipment, b) duty room for nurses and doctors, c) isolation room, d) counseling area, e) step-down area, f) equipment bay, g) breastfeeding room, h) store section, i) breast milk expression room, j) faculty room, k) handwashing areas

2. Area Preparation and Infection Control

Area preparation started with cleaning the ward with a water and soap solution, followed by a standard disinfection solution. Pest control of wooden panels and a thorough dusting of the walls and panels were done. The inner part of the ward, which functioned as a high dependency unit before shifting, was fitted with extra central gas lines to connect ventilators. Extra switchboards were installed. All electric connections and pipelines were rechecked for functionality. This was followed by fumigation and the ward was closed for use for two days before shifting. A team of staff from the infection control committee of the institute overviewed the work.

3. Route Planning

The shortest, safest, and most uncluttered route was identified, the floor was checked for evenness, and a dry run was done with a non-functional warmer for estimating the time and ease of movement. A grill gate was present on the way, which was identified as the source of a sudden jerk, and it was decided to slightly lift the warmer there, rather than filling the grill, which would have been cumbersome. The dry run revealed that the distance could be covered in four minutes.

4. Attendant Preparation

The attendant's preparation was done, as relocation of the neonates to a new place was expected to create some amount of anxiety among parents. Relatives of the admitted neonates were informed about the need and proposed day of shifting. This was done over three days after each counseling session, in the morning, which is standard practice at our center. All their concerns were addressed, and it was informed that one of the relatives would accompany the neonate at the time of shifting. All of them agreed voluntarily to the shifting.

5. Room Preparation

On the day of shifting, halogen heaters (11 in number) were used to preheat the relocation area. They were switched on, and the room was preheated to a temperature of 28ºC from 18 ºC, which took three hours.

6. Preparation of the Babies

All neonates were fed and medications administered as per the schedule and no changes in timing were made before shifting. The nurse assigned to a particular neonate prepared the neonate by covering them in cotton and gauge followed by tucking and swaddling with a blanket. This was done as no incubators or transport incubators were available in the unit. The bassinet of the warmer was covered with cling wrap and a small opening was left for air circulation. Term and preterm babies were prepared similarly.

7. Team Preparation

One consultant was placed in the NICU and one consultant in the new area before shifting, for overview and support. Five residents posted in the department were assigned the neonates to be shifted. The team for each neonate consisted of nursing staff, a resident, housekeeping staff, and a relative.

A biomedical engineer and a team of electricians were made available at the new site for maintenance of the equipment and circuit as required. A member of the hospital infection control committee was also present for monitoring. A security guard was stationed at the gate who noted the time and sequence of shift out of the neonates and entry to the proposed route was closed for movement of the public for the duration of shifting.

8. Sequence of Shifting

All store items and consumables that were thought to be necessary were shifted first. All equipment that was not currently in use but necessary was shifted after that. The feed preparation area was identified and the required equipment and containers were shifted and readied for use. A mini nursing station was established inside the new area. The neonates were shifted last.

9. Shifting of Neonates

A multipara monitor was attached to each neonate, relatives were informed and readied, the team for the baby assembled, pre-transfer vitals were recorded, warmer switched off, plug removed from the supply, and the neonate was wheeled out of the NICU on the warmer; the warmer was moved slowly with the neonate on board. The timings of leaving the NICU and of receiving in the new area were noted. This was achieved by time-matching the watches of guards posted at the exit point and a resident doctor present at the entry point. The warmer was attached to the power outlet and switched on, and post-transfer vitals were noted. The neonate was uncovered and re-examined for hemodynamic stability and signs of stress.

10. Shifting of Neonate on Ventilator Support

One neonate was on a ventilator at the time of shifting. As there was no transport ventilator with neonatal mode available in the institute, it was decided to shift the baby on flow inflating oxygen support. The neonate was similarly prepared as other babies for transport. One consultant, along with a resident and nursing staff caring for the baby, constituted the team. The neonate was disconnected from the ventilator and started on manual breaths with a flow-inflating bag. As a blender was not available, the neonate received a fraction of inspired oxygen (FiO2) of 100% during the period of transport. The ventilator was shifted and restarted with the same settings in the new area, and its functionality was confirmed telephonically to the transport team. The neonate was wheeled out of the NICU like others on the Bain circuit and reconnected to the ventilator upon arriving at the new area and the warmer plugged in and switched on.

## Results

Demographic parameters are illustrated in Table 1. A total of 16 neonates were transferred, out of which 11 were male and five were female. The mean age of the neonates was 11±9 days with a range of one to 30 days. Inborn and outborn neonates were equal in distribution (50% each). The majority (56.25%) of the neonates were born vaginally. The mean gestational age of the neonates was 35±3.49 weeks. The average duration required for the transport of each neonate was 5.62±3.03 minutes (range 2-15 minutes). The distance of travel was estimated to be about 100 meters.

**Table 1:** Demographic details

Sex	Male	11 (68.75)
	Female	5 (31.25)
Age in (days) at admission n ± SD (min-max)	2.5±2.44 (1-8)	
Age in (days) at transport n ± SD (min-max)	11±9.5 (1-30)	
Birth weight in grams (min-max)	2259±875.3 (890- 4255)	
Place of delivery	Inborn	8 (50)
	Outborn	8 (50)
Mode of delivery	Vaginal	9 (56.25)
	Caesarean	7 (43.75)
Gestational age in completed weeks n ± SD (min-max)	35±3.49 (27-39)	
Mean duration of travel in minutes n ± SD (range)	5.62±3.0304 (2-15)	

The primary diagnosis of the neonates shifted is depicted in Table 2. Sepsis (3.25%) was observed to be the most common diagnosis observed, followed by birth asphyxia and hyaline membrane disease.

**Table 2:** Diagnosis of neonates HMD: hyaline membrane disease

Primary Diagnosis	Number n(%)
HMD	3 (18.75)
Sepsis	5 (31.25)
Birth asphyxia	3 (18.75)
Apnoea of prematurity	1 (6.25)
Symptomatic polycythemia	1 (6.25)
Neonatal hyperbiirubinemia	1 (6.25)
For observation	2 (12.5)

The pre-transport and post-transport vitals are depicted in Table 3. There was a statistically significant increase in the heart rate of neonates on arrival (p = .00769). Though it was statistically significant, there was no tachycardia requiring intervention. There was a drop in the skin temperature of neonates on arrival at the new area, which was not statistically significant. The lowest recorded temperature on arrival was 36.5ºC.

**Table 3:** Vitals of the neonates

Vitals	Pre transport (mean±SD)	Post transport (mean±SD)	p-value
Temperature ºC	37.04±0.36	36.94±0.32	.33757
Respiratory rate (breaths/min)	54.44±9.51	54±11.01	.55797
Heart rate beats/min	137.7±8.51	141.3±9.01	.00769
Spo2 %	96.81±1.64	97.25±1.43	.16845
Capillary refill time in seconds	< 2	<2	

For the neonate on ventilator support, the neonate was on the synchronized intermittent mandatory ventilation (SIMV) mode of ventilation with peak inspiratory pressure (PIP) of 18, positive end-expiratory pressure (PEEP) of 5, and FiO2 of 30%; the multipara monitor malfunctioned midway. Upon arrival, the baby had a decreased SpO2 of 87% and was reconnected to the ventilator. The ventilator settings were modified, and FiO2 increased to 40%. The FiO2 increased to 92% in 2 minutes. A careful watch was maintained and there was no subsequent deterioration in the neonate.

As seen in Table 4 listing the outcome analysis out of the 16 neonates, 12 (75%) were discharged successfully. Three (18.75%) left against medical advice at a later date and one (6.25%) expired; the cause of which was late-onset sepsis. The average duration of stay of this cohort was 14±9 days.

**Table 4:** Outcome data LAMA: leave against medical advice

Outcome	Numbers n(%)
Discharged	12 (75.0)
LAMA	3 (18.75)
Expired	1 (6.25)
Average age at outcome (in days)	14 ±9

## Discussion

Various models of pre-transport stabilization are available such as STABLE (Sugar, Temperature, Airway, Blood pressure, Laboratory workup, and Emotional support) [4], SAFER (Sugar, Arterial circulatory support, Family support, Environment, and Respiratory support) [5], and TOPS Temperature, Oxygenation (Airway & Breathing), Perfusion, Sugar) [6].

All these models, in various aspects, recommend that neonates should be stabilized before the initiation of transport. If a neonate is not stabilized before transport, the outcome remains poor. A stabilized neonate can be transported to a relatively long distance safely [7]. In their study, Pai et al. came to the conclusion that longer transport periods are usually associated with a risk of deterioration but if the transport referral call was made late and the neonate was managed and stabilized, there was a lower likelihood of clinical deterioration during transport [8].

Ashokcoomar et al., in their explorative study, found that during emergency transports, lack of sophisticated equipment and malfunctioning of equipment posed an additional threat to the safe transfer of the neonates [9]. In this cohort, one of the critically ill neonate’s monitoring equipment malfunctioned midway and the deterioration could not be picked up early, which may result in poor outcomes if the transport duration is longer. One may consider carrying additional handheld smaller devices as backup during such transport.

Neonatal transport remains one of the most stressful events of neonatal care. There is always a risk of deterioration of the neonate during transport. Moving the neonate out of the open care system to the incubator may result in loss of intravenous line or endotracheal tube displacement, which was not observed in the index neonates because the neonates were moved in their warmers. Shah et al., in their study, have demonstrated that movement of the head of a neonate and endotracheal tube occurs in interhospital transport in an ambulance and inside the incubator in intrahospital transport and recommend using a gel pillow during transport [10].

Sound and vibration levels may vary during transport and may have an impact on the physiological stability of the neonate during transport. Karlson et al., in their study, concluded that the sound and whole-body vibration levels during transport result in a change in heart rates [11]. Whole-body vibration results in a lower heart rate while increased sound levels result in a higher heart rate. But in a recent study, Baily et al. concluded that during air and surface transport of neonates, though the sound and vibration levels are usually higher than the published recommendations, they do not affect the physiological stability of the neonates [12]. In the present cohort, we did not access the sound and vibration levels, but the neonates had a significantly higher heart rate at the receiving end and may have been exposed to higher sound levels during transport.

Most of the studies on neonatal transport have revealed the risk of hypothermia and hypoglycemia to be the commonly encountered complications [13-14]. The transport of neonates in our settings was done in December when the average daytime temperature remains about 23ºC. This cohort of neonates did not develop statistically significant hypothermia, but the mean temperature post transport was lower. This may be because of the short transport duration and adequate planning. The use of cling wraps over the neonate further prevented any heat loss. As there was no clinical suspicion of hypoglycemia post transport and considering the fact that the duration of transport was short, blood glucose estimation was not done on arrival. Increased duration of transport results in more clinical complications. Clinical deterioration occurred in 6.25% of neonates in the present cohort, which is lower than that of previous reports; this may be because the majority of neonates were relatively stable at the time of transport and the duration of transport was short.

Twenty-eight percent (28%) of the neonates required cardiopulmonary resuscitation on arrival as reported in a study of 50 neonates who were transferred to tertiary care in Jamaica [15]. Half of these neonates experienced cardiopulmonary deterioration during the journey, and 61% of the neonates who died required cardiopulmonary resuscitation on arrival. Also, a lack of trained personnel and lack of proper equipment was noted in the study. This implies that heart rate must be stable before and monitored during transfer. In our study, a statistically significant change was observed in the heart rate upon arrival. This could be because of the stress of transport.

During longer transport in resource-poor settings, a kangaroo mother can be used as an effective way to keep the neonate warm; it also results in stable vitals during transport as observed by Dehghani et al. [16].

Neonatal transport is also a period of stress for the parents; understanding their concerns should also be part of the process. Effective communication and the involvement of the parents result in a smoother transport process [17]. In this cohort, all the parents were informed and counseled in a phased manner which resulted in their cooperation and they became part of the process.

Mass transport of neonates may be required in events such as a fire in the unit or any other natural or man-made disasters. It may also be required for upscaling of infrastructure or change in the layout of existing facilities. For disaster-prone regions, such as Uttarakhand, one should always be prepared for emergency mass neonatal transport. Every newborn care center should have an emergency neonatal transport plan in case of a disaster. Neonatal transport is highly specialized with respect to the team, network, and equipment and the capability of quickly transferring the most critical newborn safely to an appropriate medical center must exist [18]. Also, a regular simulation of different disaster scenarios should be done, as constant practice will improve emergency plans [19]. With the development of communication and social networking, a constant communication channel can be established between the referring and the receiving units, which may be conducive to the safety of neonates during transport [20]. Also, post the transport of a large number of neonates, the continuation of the patient services at the new site requires planning, leadership, patience, flexibility, and commitment from all stakeholders, which were observed in disaster-affected NICU [21-22]. Even though the transport in the present study was a planned activity, it lead to physiological destabilization of the neonates and unforeseen events, such as equipment malfunction, which emphasizes the need to be vigilant and ready for such scenarios.

There are certain limitations to this study. Outcome data of all neonates were not complete, as three neonates went against medical advice and could not be followed up. The transport was of very short duration and thus the effect of transport of longer duration during such an exercise could not be accessed. Also, as this cohort of neonates were transported intramurally, the findings cannot be extrapolated to out-of-hospital transport scenarios.

## Conclusions

Adequate planning and a trained neonatal transport team can decrease adverse outcomes during neonatal transport. Mass transport of neonates may be required during disasters and accidents such as fires. Transport is a period of stress to the neonates. Prior planning, a written action plan, and regular drill of involved staff can limit the adverse outcomes and deterioration of neonates during such transport.
